# Supplementary material for: HSV1716 Prevents Myeloma Cell Regrowth When Combined with Bortezomib In Vitro and Significantly Reduces Systemic Tumor Growth in Mouse Models
Source: Viruses. 2023 Feb 22;15(3):603. doi: 10.3390/v15030603 (PMC10059747; doi:10.3390/v15030603)
Supplement: Supplementary file 1 [file viruses-15-00603-s001.zip › viruses-2218930-supplementary.pdf]

Supplementary Materials

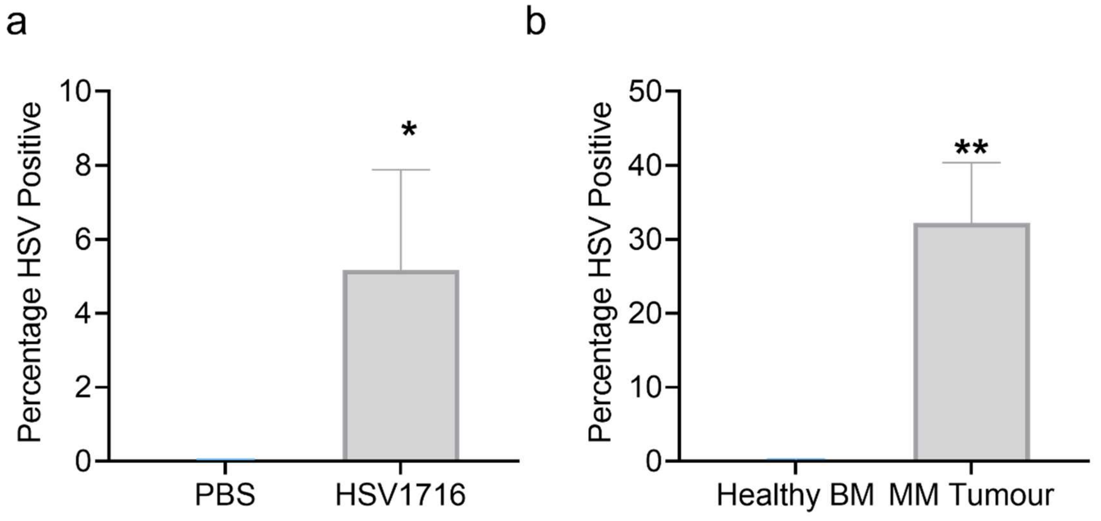

Figure S1. HSV1716 particles found only in Tumour areas of HSV1716 treated xenograft mice. At sacrifice tibial bones were fixed and decalcified before paraffin sections were stained for HSV viral particles. **(a)** Percentage of HSV1716 staining in PBS and HSV1716 treated mice. Data are mean ± SEM, n=5 mice per group. * = $p < 0.05$ unpaired T-test, w.r.t control. **(b)** Percentage of HSV1716 particles within JJN-3 tumour areas and healthy bone marrow areas within HSV1716 treated mice. Data are mean ± SEM, ** = $p < 0.01$unpaired T-test, w.r.t control.

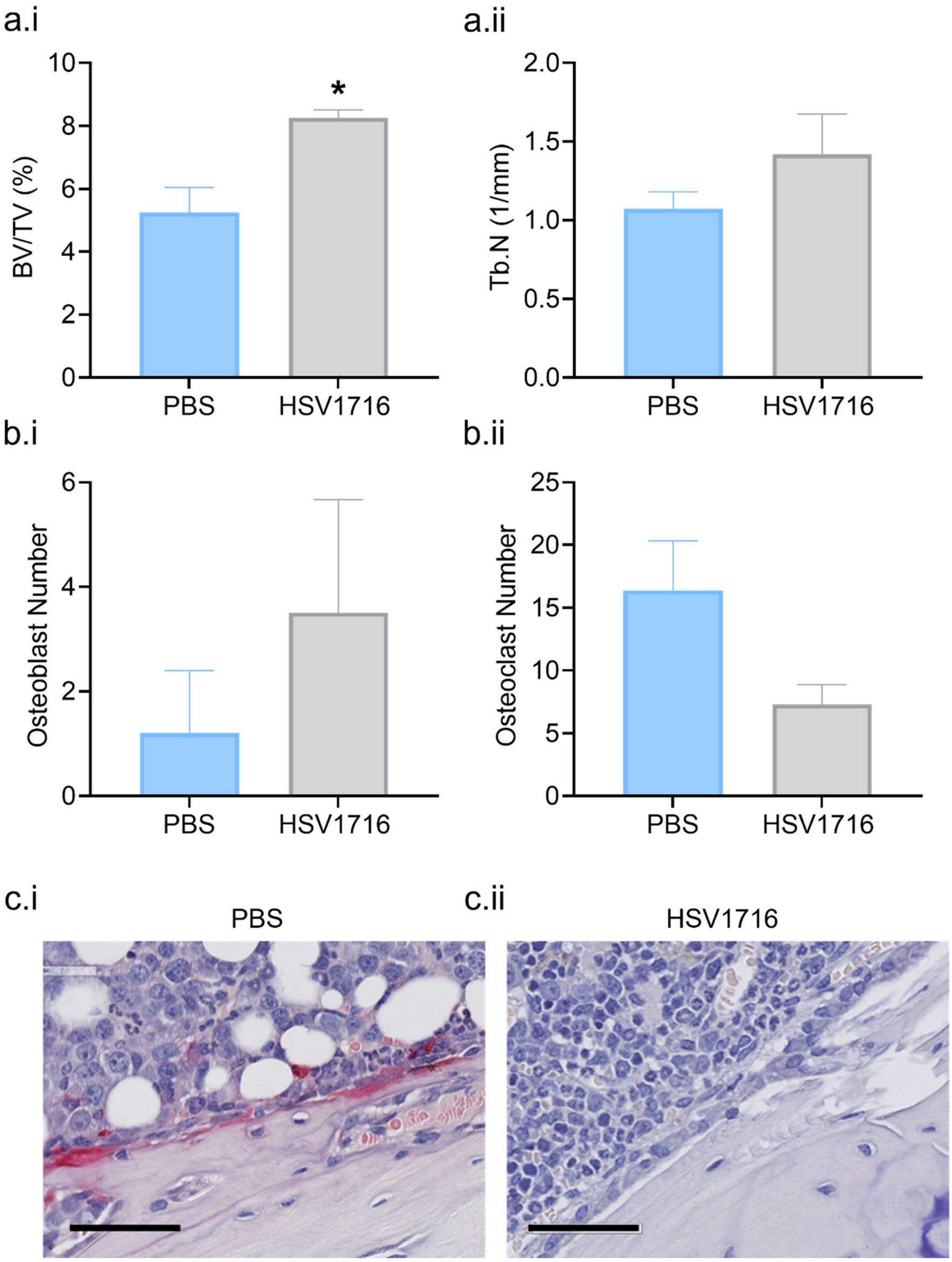

Figure S2. HSV1716 treated xenograft mice had increased trabecular volume. At sacrifice tibial bones were fixed and scanned by micro-CT to assess myeloma-induced bone disease. **(a.i)** Trabecular volume as assessed as a percentage of bone volume/trabecular volume in PBS or HSV1716 treated mice. **(a.ii)** Trabecular number in PBS or HSV1716 treated mice. Data are mean ± SEM, n=5 mice per group. * = p <0.05 unpaired T-test, w.r.t control. Following micro-CT, bones were decalcified and paraffin embedded. Bone histomorphometry was performed in TRAP stained sections. **(b.i)** Osteoblast number in PBS or HSV treated mice, **(b.ii)** osteoclast number in PBS pr HSV treated mice. Data are mean ± SEM, n=5 mice per group. * = p < 0.05 unpaired T-test, w.r.t control. Representative images of TRAP positive osteoclasts (stained red) on the cortico-endosteal surface of tibiae from (c.i) PBS treated mice and **(c.ii)** HSV1716 treated mice, Bar = 100 μm.

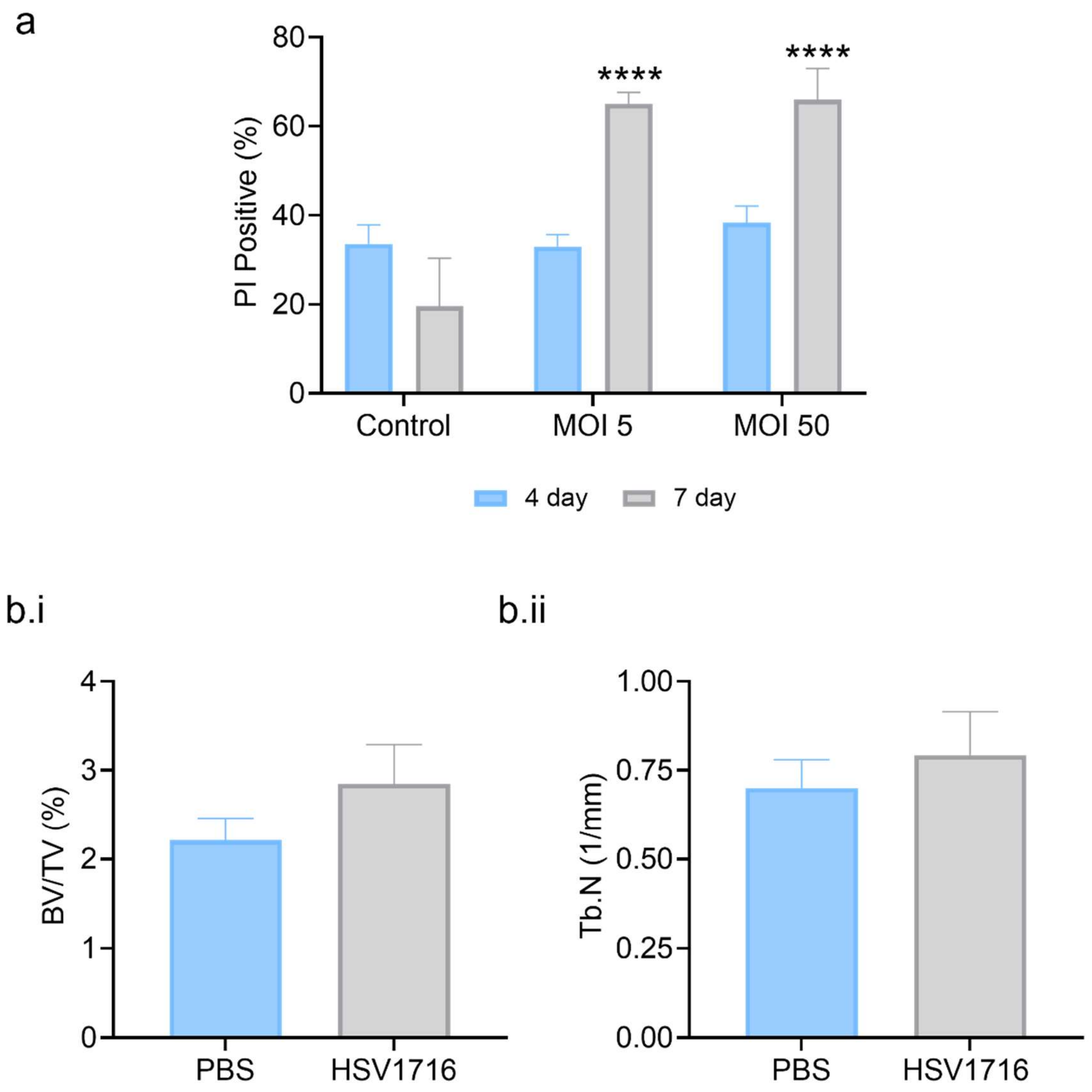

Figure S3. HSV1716 induces delayed cell death *in vitro* in 5TGM1 cells and does not significantly alter trabecular bone volume in 5TGM1 syngeneic *in vivo* model. (a) 5TGM1 cells were treated with HSV1716 at MOI 5 or 50. After 4 and 7 days, cell death was assessed by PI staining and flow cytometry. Data are mean ± SEM, n=3. * = $p < 0.05$ 2way ANOVA. At sacrifice tibial bones were fixed and scanned by micro-CT to assess myeloma-induced bone disease. (a.i) Trabecular volume as assessed as a percentage of bone volume/trabecular volume in PBS or HSV1716 treated mice. (a.ii) Trabecular number in PBS or HSV1716 treated mice. Data are mean ± SEM, n=5 mice per group. **** = $p < 0.0001$ unpaired T-test, w.r.t control.

Table S1. SYBR Green Primer Sequences.

Gene	Forward	Reverse
**ICP0**	5' AAGCTTGGATCCGAGCCCCGCCC	5' AAGCGGTGCATGCACGGGAAGGT
**ICP8**	5' GACATTACGTTCACGGCCTTCGAAGCCAG	5'GGCCGAGTTGGTGCTAAATACCATGGC
**GAPDH**	5' TGCACCACCAACTGCTTAGC	5' GGCATGGAC TGTGGTCATGAG
